# Abnormal expression of TSG-6 disturbs extracellular matrix homeostasis in chondrocytes from endemic osteoarthritis

**DOI:** 10.3389/fgene.2022.1064565

**Published:** 2022-11-18

**Authors:** Yujie Ning, Pan Zhang, Feiyu Zhang, Sijie Chen, Yanli Liu, Feihong Chen, Yifan Wu, Shujin Li, Chaowei Wang, Yi Gong, Minhan Hu, Ruitian Huang, Hongmou Zhao, Xiong Guo, Xi Wang, Lei Yang

**Affiliations:** ^1^ Key Laboratory of Trace Elements and Endemic Diseases, School of Public Health, Xi’an Jiaotong University Health Science Center, National Health Commission of the People’s Republic of China, Xi’an, China; ^2^ Sichuan Center for Disease Control and Prevention, Chengdu, China; ^3^ Foot and Ankle Surgery Department, Honghui Hospital of Xi’an Jiaotong University, Xi’an, China; ^4^ Clinical Research Center for Endemic Disease of Shaanxi Province, the Second Affiliated Hospital of Xi’an Jiaotong University, Xi’an, China; ^5^ School of Nursing, Health Science Center, Xi’an Jiaotong University, Xi’an, China

**Keywords:** Kashin-Beck disease, chondrocytes, TSG-6, HT-2 toxin, extracellular matrix

## Abstract

**Background and aims:** Kashin-Beck disease (KBD) is a unique endemic osteochondropathy with unclear pathogenesis in China. T-2 toxin exposure has been identified as a significant risk factor of KBD. However, the mechanism of articular cartilage damage induced by T-2 toxin is a conundrum. We explored the role of the extracellular matrix-related gene TSG-6 in the articular chondrocyte damage process under the exposure of HT-2 toxin.

**Methods:** TSG-6 was identified as a candidate gene by mining our previous gene expression profiling of KBD and verified by qRT-PCR and immunohistochemistry. Then, TSG-6 was silenced by RNA interference technology and overexpressed induction by TNF-α. Gradient concentrations of HT-2 toxin were added to intervene with C28/I2 chondrocytes. MTT was used to observe the proliferation and cell viability of chondrocytes, and qRT-PCR was utilized to detect the expression changes of MMP1, MMP3, MMP13, COL2A1, and proteoglycan before and after treatments for verification.

**Results:** TSG-6 was upregulated in KBD chondrocytes at the mRNA level and upregulated in the superficial, middle, and deep zones of KBD cartilage. After TSG-6 silencing, the expression of MMP1, MMP3, MMP13, and proteoglycan was significantly decreased while COL2A1 expression was significantly increased, which was reversed after the overexpression of TSG-6 induced by TNF-α (*p* < 0.05). The survival rate of chondrocytes was correspondingly reduced with an increase in the HT-2 toxin concentration. Compared with the blank control group, the expression of MMPs was increased in the intervention group of HT-2 toxin, while the expression of proteoglycan and COL2A1 decreased (*p* < 0.05).

**Conclusion:** The upregulation of the TSG-6 gene may play a role in promoting the damage and degradation of the extracellular matrix in KBD chondrocytes under the exposure of HT-2 toxin.

## Introduction

Kashin-Beck disease (KBD) is an endemic osteochondropathy found in China, southeastern regions of Transbaikalia in Russia, and North Korea, and sporadic cases are found in Central Asia and the European part of Russia (Ermakov et al., 2019). It is characterized by childhood onset, arthritic pain, enlarged joints, limited mobility, and dwarfism in advanced patients (Han et al., 2018). The clinical manifestations and pathological features of KBD are similar to those of OA such as joint pain, stiffness, limitation of motion, swelling, chondrocyte necrosis, apoptosis, and degradation of the extracellular matrix (ECM) (Wang et al., 2017). Although the etiology and pathogenesis for KBD still need to be elucidated, cereal T-2 toxin exposure has been identified as an important risk factor of KBD (Sun et al., 2019). However, the mechanism of articular cartilage damage induced by the T-2 toxin is unclear.

TSG-6, which is also known as TNFAIP6, encodes a secreted protein that contains a hyaluronic acid-binding domain; therefore, TSG-6 belongs to a member of the hyaluronan-binding protein family. Previous studies determined that the hyaluronic acid domain is primarily involved in the stability of the extracellular matrix and cell migration (Dyer et al., 2016). Moreover, TSG-6 plays an important role in anti-inflammatory and cartilage protection from arthritis by forming a negative feedback loop (Wisniewski et al., 1996). In recent decades, we identified an environment-gene interaction that plays an important role in the cartilage damage process of KBD (Wang et al., 2017; Lin et al., 2019; Yang et al., 2019). For example, T-2 toxin can stimulate IL-1ß production in chondrocytes, which collectively aggravates cell damage by inhibiting the transcription of collagen II and aggrecan, suppresses the release of sulfated glycosaminoglycans (sGAG) and TIMP1, and promotes matrix metalloproteinase production including MMP1, MMP3, and MMP13 (Chang et al., 2017). Moreover, T-2 toxin combined with low-selenium administration in a rat model proved to remarkably upregulate TGF-βR-I-II proteins to activate the TGF-β signaling pathway, which plays an important role in the induction of chondrocyte death in the deep zone of articular cartilage similar to human KBD (Zhang et al., 2018).

Extracellular matrix (ECM) degradation in chondrocytes is a typical pathological abnormality of KBD (He et al., 2018). However, it is unclear whether the T-2 toxin can directly induce this degradation. Specifically, the critical intermediate element and functional pathway remain need to be elucidated. Therefore, we identified candidate T-2 toxin-responsive genes and their effect on the ECM degradation process of KBD. In this study, the comparison between the previous gene expression profiles of chondrocytes in KBD and OA articular cartilage was analyzed, and TSG-6 was identified as a target gene verified by qRT-PCR and IHC. Then, TSG-6 was silenced and overexpressed to observe the effect on the change in the ECM of chondrocytes. Finally, gradients of HT-2 toxin, the main cell-lethal metabolite of T-2 toxin after ingestion, were added to C28/I2 cells for verification.

## Materials and methods

All subjects provided informed consent (orally if the subject was illiterate) regarding the sample collection. The study protocol was approved by the ethics committee of Xi’an Jiaotong University (Approval No. 2018–206).

### Articular cartilage sample recruitment

Diagnoses and degree classifications of patients were strictly applied according to the national criteria of KBD in China [WS/T 207–2010]. All subjects with alterations, such as defects and sclerosis on the bone end of phalanges combined with compression changes of the calcaneus and talus and enlarged/deformed limb joints (e.g., hand, elbow, knee, and ankle) manifested on X-ray, were diagnosed with KBD. Subjects were excluded if they were suffering or had previously suffered from any other osteoarticular diseases (such as rheumatoid arthritis, gout, or skeletal fluorosis) or any other type of macrosomia, osteochondrodysplasia disorder, or chronic disease (such as hypertension, diabetes, or coronary heart disease) or had received any treatment in the past six months. Clinical information was collected from patient records. Articular cartilage samples from KBD and OA patients were collected from individuals who underwent arthroplasty of the knee. Healthy controls were obtained from patients who suffered trauma or amputation due to an accident.

Donors signed a written informed consent form. Subjects were of Chinese Han lineage. For the verification of TSG-6 by qRT-PCR and immunohistochemistry, specimens of adult articular cartilage were collected from five KBD patients who had arthroplasty in the knee and five healthy subjects who had an amputation due to an accident in Xi’an Red Cross Hospital (Supplementary Table S1). For silencing and overexpression of TSG-6, articular cartilage samples were obtained from five KBD patients and three controls for the same reason described above (Supplementary Table S2).

### Cartilage tissue collection and chondrocyte isolation

All articular cartilage samples, including all of the cartilage zones (including calcified) and subchondral bone, were harvested from the lateral tibial plateau and obtained within 1 h after operation. Chondrocytes were isolated as follows: articular cartilage specimens were washed twice with sterile phosphate-buffered saline (PBS) with antibiotics (penicillin and streptomycin), cut into pieces (1 mm^3^), and subjected to enzymatic digestion with 0.25% trypsin at 37 °C in an atmosphere of 5% CO_2_ for up to 30 min. Cell suspensions were centrifuged at 1,000×g for 5 min, the supernatant was completely aspirated, and the cells were digested in basal media supplemented with 0.2% type II collagenase at 37 °C using an Eppendorf Thermomixer for 12–16 h.

### Immunohistochemical verification

Cartilage tissues were fixed with 4% (w/v) paraformaldehyde for 24 h immediately after acquisition and decalcified in 10% (w/v) ethylenediaminetetraacetic acid disodium salt (EDTA-Na2) for 2–3 weeks. Samples were dehydrated in an alcohol series, cleared in xylene, and embedded in paraffin wax. Paraffin sections were cut into 5 µm sections, mounted on slides, and stored at room temperature until ready for staining. The paraffin-embedded sections were baked at 65 °C for 1 h, deparaffinized with xylene and then rehydrated in decreasing concentrations of ethanol. Endogenous peroxidase activity was blocked by 0.3% (w/v) hydrogen peroxide for 10 min at room temperature, and the sections were then washed with 1×PBS. Then, sections were incubated in a 10 mol/L urea solution diluted with water at 37 °C for 20 min and washed with 1×PBS. Sections were incubated in 0.1% trypsin/CaCl_2_ at 37 °C and digested for another 20 min for antigen retrieval. After blocking with 5% (w/v) goat serum for 20 min at room temperature, anti-TNFAIP6 (1:100 dilution, PA5-76008, Invitrogen) antibodies and IgG as a negative control were applied onto the sections, and the samples were further incubated overnight at 4 °C. After washing with 1×PBS, sections were incubated using a human serum amyloid P (SAP) kit (solution B contains a goat anti-rabbit secondary antibody; Zhongshan, Jinqiao, Guangzhou, China) according to the manufacturer’s instructions. The substrate 3,3′-diaminobenzidine was added to stain the sections, and hematoxylin counterstaining was performed. Finally, sections were dehydrated and mounted under alcohol-cleaned coverslips. All IHC staining was assessed under light microscopy by two pathologists who were blinded to the origin of the samples. Articular cartilage was divided into three cell morphologies, namely, the superficial, middle, and deep zones, according to light microscopy observation. Chondrocytes in the superficial zone were relatively small and flat and were oriented with the long axis parallel to the surface; cells in the middle zone were larger and more rounded and were randomly distributed in the matrix with fibers running in oblique directions; and cells in the deep zone were larger in size and were arranged in a columnar manner perpendicular to the surface. An assessment of staining throughout each cartilage zone included systematic counting of positive and negative cells starting from the cartilage surface and progressing down through all layers of cartilage. Five randomly chosen fields in each zone were counted at 50× magnification. The percentage of positive cells was calculated using the number of positively stained cells divided by the total number of cells (positively and negatively stained cells) in the chosen fields of view. Percentages of positive cells in different zones were calculated for each case and for the different groups.

### Candidate gene identification and verification

According to the previous gene expression profile in cartilage from KBD (Wang et al., 2009) and OA microarray data GSE57218 from the GEO database in NCBI, TSG-6 (TNF-stimulated gene 6, also known as TNFAIP6) was identified as one of the common differentially expressed genes of importance in ECM reconstruction and by maintaining cellular homeostasis. Differential expression of TSG-6 at the mRNA level in KBD chondrocytes was verified respectively by quantitative real-time polymerase chain reaction (qRT-PCR). TaKaRa Company (Japan) synthesized the TSG-6 primer (forward-GGAGTGAAAGATGGGCATGCC, reverse-CTCATTTGGGAAGCCTGGA). Experiments were conducted according to the manufacturer’s instructions.

### TSG-6 silencing and overexpression

To clarify the effect of TSG-6 abnormality in the ECM degradation of KBD chondrocytes, we silenced this gene in KBD chondrocytes by RNA interference technology and then separately overexpressed TNF-α induction. A pretest was applied to determine the optimum multiplicity of infection (MOI) and transfection time of the lentivirus. We designed five culture conditions combined with three preset MOI (Supplementary Table S3) and selected the group with an infection efficiency above 80% along with good cell growth status. The lentivirus system (GENECHEM, Shanghai) consisted of the objective vector GV248, pHelper 1.0, and pHelper 2.0. We designed three sections of TSG-6 for construction of the lentivirus vector including TNFAIP6-RNAi(54322–1), TNFAIP6-RNAi(54323–1), and TNFAIP6-RNAi(54324–11) (Supplementary Table S4). Transfected 293T cells were introduced into the lentivirus system for 48–72 h and then the supernatant was collected. Next, viruses were concentrated and purified by 0.45 μm filtration and ultracentrifugation. Finally, the quality of lentiviruses was determined involving physical form, sterility, and a titer test (*via* fluorescence).

Primarily, chondrocytes were cultured in 96-well plates (5×10^4^ cells/well) for 36 h. Then, 200 μl TNF-α (0, 5, 15, 25, 50, 75, 100 ng/ml, 5 repeats for each concentration) were added to further culture chondrocytes with 5% CO_2_ for 24 h, 48 h, and 72 h (three repeats for each time point) at 37 °C. TNF-α is not supposed to remarkably affect the proliferation of chondrocytes. We attempted cell culture with serum-free medium as described in previous studies (Zhang et al., 2012; Yoshida et al., 2018). However, a downward trend of cell proliferation was observed when the TNF-α concentration added up to 100 ng/ml by MTT. This might have been due to the serum-free culture condition. Therefore, we added 2% serum into the medium, but the proliferation still presented a downward trend at 100 ng/ml of TNF-α. Hence, 100 ng/ml was set as the upper limited concentration. Both trends emerged at 48 h. No significant difference in the affection of cell proliferation was observed between the previously mentioned concentrations, which suggests that there is no need to try so many sub-dosages. Finally, we selected four concentrations of TNF-α (0, 5, 25, 100 ng/ml) with serum-free medium and one optimistic time point (48 h) for TSG-6 induction.

qRT-PCR was used to detect the expression changes of extracellular matrix MMP1, MMP3, MMP13, COL2A1, and proteoglycan (Abcam, United Kingdom, 1:500 dilution) before and after treatments. Information about the primers (TaKaRa, Bejing) of each gene is listed in Supplementary Table S5. Proliferation and viability of chondrocytes was observed by MTT before and after the gradient concentrations of TNF-α treatment.

### HT-2 toxin intervention

To explore whether TSG-6 is the intermediate element in the chondrocyte ECM degradation process caused by T-2 toxin, gradients (0, 12, 50, 100, 150, 200, 250 nM, and 200 μl) of HT-2 toxin were added to intervene with the C28/I2 chondrocytes. Conventional culture conditions and repeat counts were the same as the overexpression section.

The morphological changes of chondrocytes were observed under a light microscope. The MTT test showed a dose-dependent inhibition effect of HT-2 on cell viability at 0, 12, 50, and 100 nM. However, the inhibitory effect did not continue to significantly increase with the increase in intervention concentration from 100, 150, and 200–250 nM. Therefore, we selected 0, 12, 50, and 100 nM to utilize in the following study.

Total RNA was extracted in subsequent experiments. The expression of TSG-6, MMPs, COL2A1, and proteoglycans in the extracellular matrix were detected using qRT-PCR before and after intervention according to previously described methods (Wang et al., 2017).

### Statistical analysis

A statistical analysis was performed using the Statistical Package for the Social Sciences for Windows version 18.0 (SPSS, Inc.). Individual samples were divided into triplicate for the study. The differences in means were determined by one-way analysis of variance (ANOVA) for multiple comparisons. Student’s t-test was applied to determine the difference between two groups. The normality and homogeneity of variance of the data were tested before any further analyses. A non-parametric test was performed if the conditions for normality and homogeneity properties were not fulfilled, and *p* values less than 0.05 were considered significant.

## Results

### Identification of upregulated TSG-6 in KBD

**FIGURE 1:**
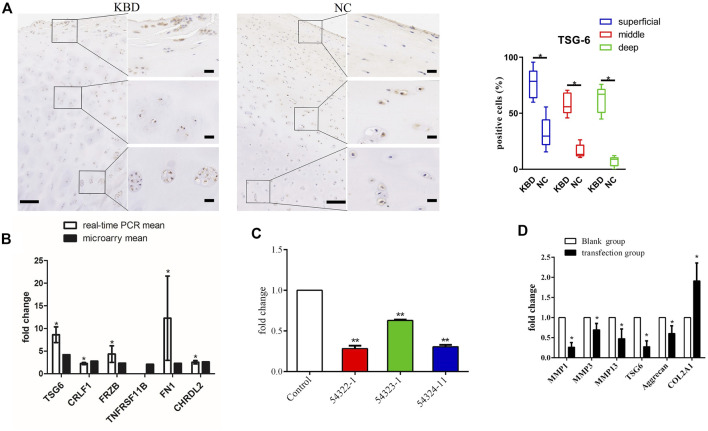
**(A)** Representative immunohistochemistry staining of TSG-6 in KBD and healthy cartilage tissues. Scale bar, left, 500 μm; right, 50 μm, and comparative quantification of positive cells of different areas (superficial, middle, and deep) between KBD and healthy cartilage tissues displayed by box plot (*n* = 3). *p < 0.05. **(B)** The mRNA levels of the selected genes between the microarray and quantitative RT-PCR in chondrocytes from patients with KBD (*n* = 3) and normal controls (*n* = 3). The histogram shows the fold change of selected genes as measured by microarray and quantitative RT-PCR; * indicates *p* < 0.05. **(C)** The selection of an optimistic transfection target for TSG-6, and the interference effect of TSG-6RNAi target sequence interfered with RNAi and detected by quantitative RT-PCR; ** indicates *p* < 0.001. **(D)** The mRNA levels of MMP1, MMP3, MMP13, aggrecan, and COL2A1 after silencing TSG-6; * indicates *p* < 0.05.

We used immunohistochemistry to detect TSG-6 protein expression in KBD cartilage tissue (Figure 1A). Results showed that TSG-6 was upregulated in the superficial, middle, and deep zones of KBD cartilage compared to cartilage tissue from normal controls. We validated the gene expression of TSG-6 in patients with KBD using qRT-PCR and found that the results were consistent with those of the differentially expressed TSG-6 gene in KBD chondrocytes screened by previous microarray analysis (Figure 1B).

### Upregulated TSG-6 was responsible for ECM degradation in KBD chondrocytes

The lentiviral transfection pretest determined that the optimal transfection conditions were MOI = 100, ENi.S + P(E)+virus for culture, and 96 h for the duration. TNFAIP6-RNAi (54322–1) had the best infection efficiency and showed a 72% interference rate (Table 1; Figure 1C). After TSG-6 silencing, the expression levels of MMP1, MMP3, MMP13, and proteoglycan were significantly decreased, while the expression of COL2A1 was significantly increased in KBD chondrocytes (*p* < 0.05, Figure 1D).

**TABLE 1:** Determination of the interference rate by qRT-PCR.

TSG-6 gene sections	Fold change	Interference rate (%)	*p* Value
Control	1	—	-
TSG-6-RNAi (54322–1)	0.28	72	<0.001
TSG-6-RNAi (54323–1)	0.63	37	<0.01
TSG-6-RNAi (54324–11)	0.31	69	<0.001

**FIGURE 2:**
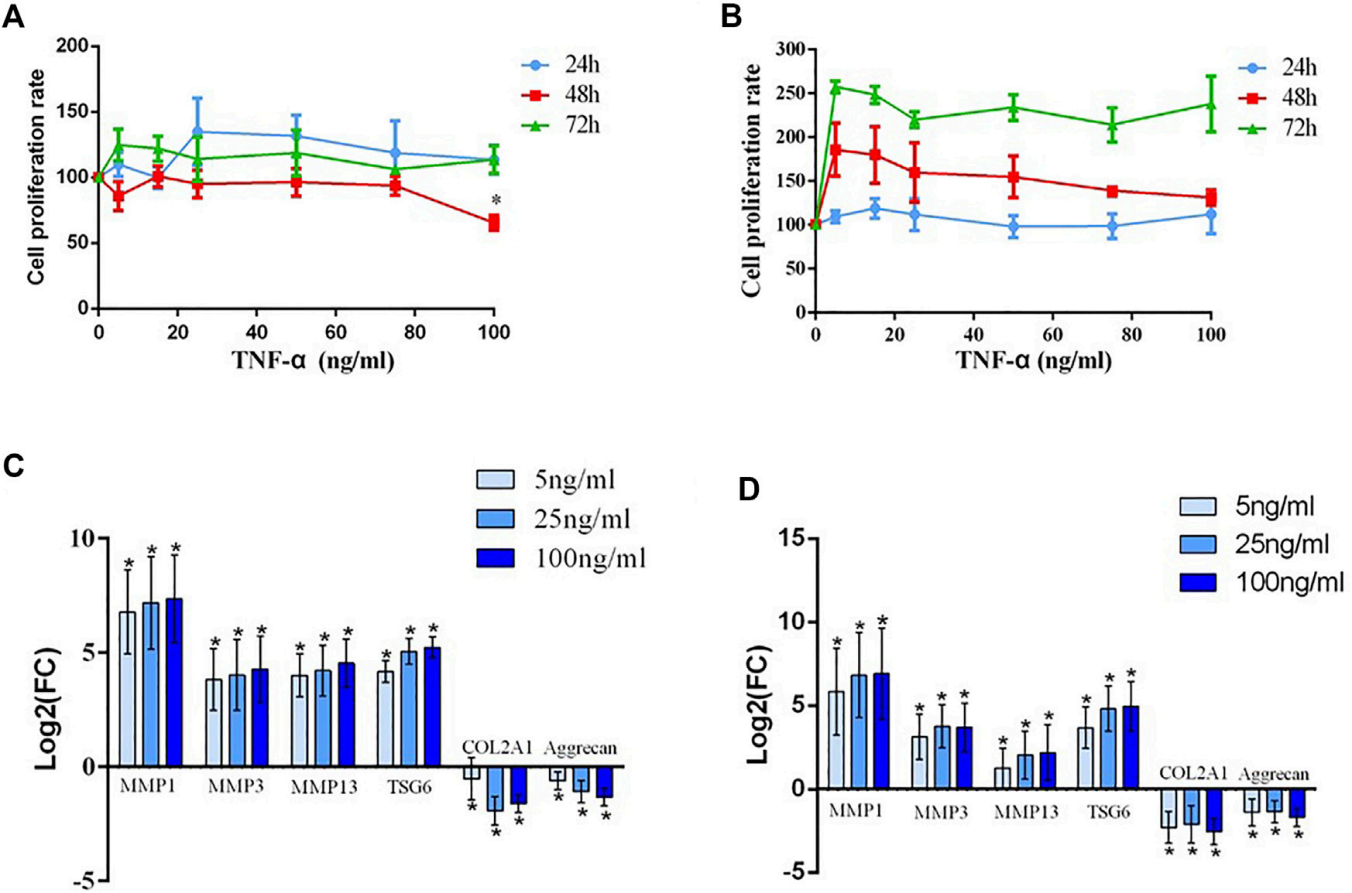
**(A)** The detection of the human chondrocyte proliferation rate after treatment with different concentrations (20, 40, 60, 80, and 100 ng/ml) of TNF-α and without serum for 24 h, 48 h, and 72 h; * indicates *p* < 0.05. **(B)** The detection of the human chondrocyte proliferation rate after treatment with different concentrations (20, 40, 60, 80, and 100 ng/ml) of TNF-α and with serum for 24 h, 48 h, and 72 h. **(C)** The mRNA expression of MMP1, MMP3, MMP13, TSG-6, COL2A1, and aggrecan after chondrocytes from patients with KBD were interfered with TNF-α; * indicates *p* < 0.05. **(D)** The mRNA expression of MMP1, MMP3, MMP13, TSG-6 COL2A1, and aggrecan after chondrocytes from normal controls were interfered with TNF-α; * indicates *p* < 0.05.

Generally, with increasing TNF-α concentration and prolonged time, cell proliferation was faintly affected regardless of whether the chondrocytes were cultured with or without serum. However, cell proliferation showed a decreasing trend at 48 h and a significant difference at a dosage 100 ng/ml without serum compared to the blank group (*p* < 0.05) (Figure 2A). And cell proliferation showed no significant difference when cultured with serum (Figure 2B).

Before intervention, the expression levels of MMP1, MMP3, and MMP13 in the KBD group notably increased, while the expression levels of COL2A1 and proteoglycan significantly decreased compared with those in the healthy control group. TNF-α intervention results demonstrated that the expression of TSG-6 increased in KBD and healthy chondrocytes in a dose-dependent manner. Increased MMPs and decreased COL2A1 and proteoglycan were more significant in TNF-α treated KBD chondrocytes than in the blank KBD group (*p* < 0.05, Figure 2C). Moreover, healthy chondrocytes treated with TNF-α also had significantly increased MMPs and decreased COL2A1 and proteoglycan compared with the blank control group (*p* < 0.05, Figure 2D).

### TSG-6 may be an intermediate factor in the chondrocyte ECM degradation process caused by HT-2 toxin

**FIGURE 3:**
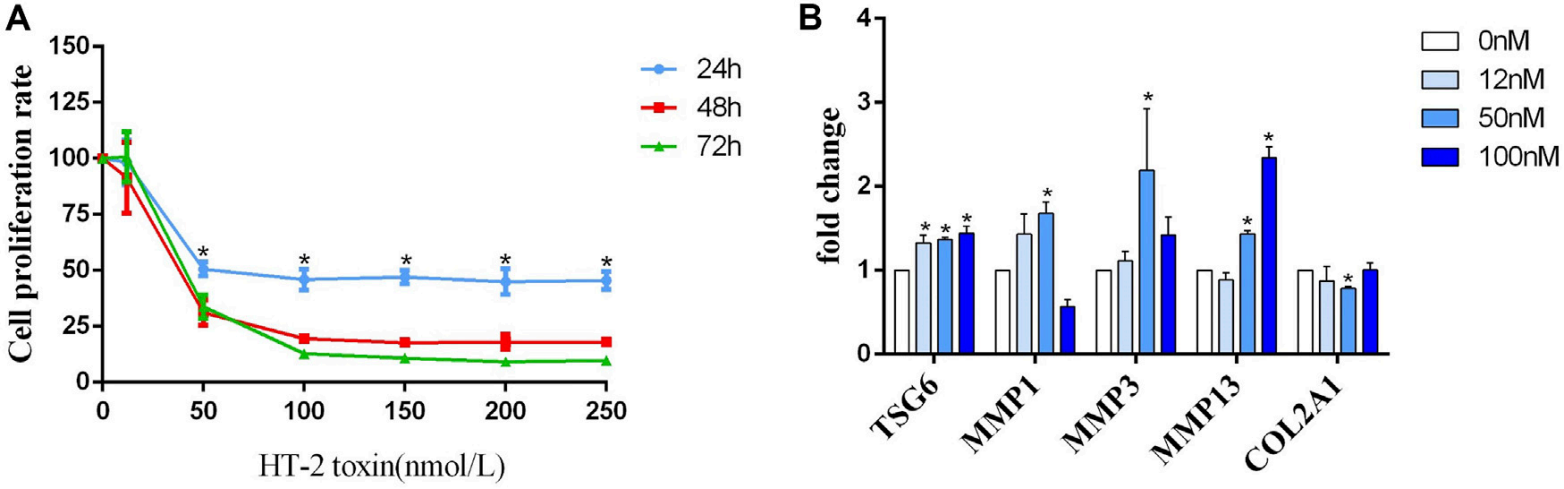
**(A)** The detection of the human C28/I2 cell proliferation rate after treatment with different concentrations (0, 50, 100, 150, 200, and 250 nmol/L) of HT-2 toxin for 24 h, 48 h, and 72 h; * indicates *p* < 0.05. **(B)** The mRNA expression of MMP1, MMP3, MMP13, TSG-6, and COL2A1 after human C28/I2 cells were treated with different concentrations (0, 12, 50, and 100 nmol/L) HT-2 toxin; * indicates *p* < 0.05.

C28/I2 chondrocytes were treated with gradient concentrations of HT-2 toxin to clarify whether TSG-6 mediates environmental exposure and chondrocyte ECM degradation. By using MTT and light microscopy, we found that the survival rate of chondrocytes was correspondingly reduced in a dose-dependent manner (Figure 3A). Compared with the blank control group, the mRNA levels of TSG-6, MMP1, MMP3, and MMP13 were increased in the HT-2 toxin intervention group (0, 12, 50, and 100 nM), while the expression of COL2A1 was significantly decreased (*p* < 0.05, Figure 3B).

## Discussion

For the first time, this study found that TSG-6 was upregulated in KBD chondrocytes, which may function as an intermediate factor between HT-2 toxin exposure and cartilage injury by promoting the ECM degradation of chondrocytes.

TSG-6 is a member of the hyaluronan (HA) binding protein family and participates in maintaining ECM stability and cell migration (Wisniewski et al., 2014). This gene is described as a double-edged sword for OA. On one hand, it could combine with aggrecan and chondroitin-4-sulfate to inhibit inflammation and protect against articular cartilage. TSG-6 reduces bone resorption activity and promotes the formation of osteophytes that contain both bone and cartilage (Broeren et al., 2015). Link_TSG6 and rhTSG-6 proteins protect cartilage from degradation by suppressing the response of MSC-derived chondrocytes to inflammatory cytokines (IL-1 and TNF) and inhibiting their expression of ADAMTS4, ADAMTS5, and MMP13 (Day et al., 2016). On the other hand, TSG-6 in intermediate and deep regions of OA cartilage could block matrix assembly directly or indirectly through activation of matrix metalloproteases (MMPs), such as MMP3, MMP9, MMP12, and MMP13, and therefore promote the progression of cartilage damage (Chou et al., 2018). Therefore, TSG-6 activity was proposed as a promising independent biomarker for OA progression (Wisniewski et al., 2014). However, determining whether TSG-6 is a protective factor or a risk enhancer of KBD development needs to be explored.

We observed decreased MMP1, MMP3, and MMP13 along with increased COL2A1 after TSG-6 silencing *via* lentivirus in KBD chondrocytes. In contrast, overexpression of TSG-6 increased MMPs and decreased aggrecan and COL2A1 in a dose-dependent manner. These findings indicate that TSG-6 upregulation in KBD chondrocytes could disturb ECM homeostasis and accelerate the cartilage damage process.

KBD is a degenerative osteochondropathy that manifests with excessive apoptosis and ECM degradation. The association between T-2 toxin and the occurrence of KBD was established by Professor Yang in the 1990s based on an epidemiological investigation and *in vitro* research (Jianbo, 1998). Evidence showed that T-2 toxin aggregated excessively in cereals obtained from endemic areas compared to nonendemic areas (Jianbo et al., 1995). Chondrocytes treated with T-2 toxin upregulated matrix metalloproteinases including MMP1, MMP3, and MMP13 and downregulated aggrecan and COL2A1 at both the mRNA and protein levels (Zhang et al., 2010; Lu et al., 2012). However, we scarcely detected T-2 toxin in patient serum and chondrocytes, which led us to deliberate on the functional form of this pathogenic factor. In a rat model and one hour after consumption, T-2 toxin mainly transformed into HT-2 toxin and was distributed in multiple organs. HPLC-MS/MS determination results showed that the order of concentration of HT-2 was as follows: femur > knee joint > rib cartilage > liver > skeletal muscle > heart > kidney. The conversion ratio occurred in the range of 68.2–90.7% (Yu et al., 2017b). In the T-2 toxin treated chondrocytes, after 48 h, the concentration of T-2 decreased from 20 ng/ml to 6.67 ± 1.02 ng/ml, while the concentration of HT-2 increased from 0 ng/ml to 6.88 ± 1.23 ng/ml (Yu et al., 2017a). This suggests that T-2 toxin was almost completely metabolized into HT-2 at the end of 48 h. Additionally, it is suggested that both T-2 toxin and HT-2 toxin can lead to apoptosis and autophagy in human chondrocytes (Yu et al.,2019b). Hence, this study discovered the effect of HT-2 toxin on the ECM degradation of KBD.

The MTT assay revealed that HT-2 could reduce the proliferation of C28/I2 cells in a dose- and time-dependent manner, which was consistent with results induced by the T-2 toxin. What attracted our attention was that cell viability did not further decrease any time point when the HT-2 concentration reached 100 nM. Therefore, the cytotoxic effect of the HT-2 toxin on chondrocytes may have a threshold.

Compared with the blank control group, the expression of TSG-6 along with MMP1, MMP3, and MMP13 was upregulated in the HT-2 toxin intervention group in a dose-dependent manner, while the expression of proteoglycan and COL2A1 significantly decreased. Therefore, based on the effect of TSG-6 on accelerating ECM degradation in KBD chondrocytes as proven by viral silencing and TNF-α induction, TSG-6 might work as an intermediate factor between HT-2 exposure and cartilage damage in the development of KBD.

During developmental and inflammatory processes, heavy chains (HCs) from the serum-derived proteoglycan inter-α-inhibitor (IαI) are covalently transferred to HA *via* the TSG-6 enzyme to form an HC-HA complex, which is similar to osteoarthritis and rheumatoid arthritis (Lauer et al., 2015; Torihashi et al., 2015). In the transfer action, HA substitutes for the chondroitin sulfate (CS) chain on bikunin to bind to the HCs *via* an ester bond. Although bikunin and its CS chain are not components of HA-HC complexes, they are integral for HA-HC complex formation (Lord et al., 2013). The organized pericellular HA coat is anchored to its principal cell surface receptor cluster of differentiation 44 (CD44). TSG-6-dependent HC-HA interactions induce the formation of a pericellular HA matrix, which facilitates CD44 relocalization within the cell membrane. This receptor complex then activates the downstream signaling cascade that is responsible for reorganization of the actin cytoskeleton (Martin et al., 2016). Significantly elevated levels of HA and CD44 were observed in the serum of both juvenile and adult KBD patients compared to healthy controls in non-KBD areas (Cao et al., 2008; Yu et al., 2014). T-2 toxin was identified as responsible for increased CD44 and HA in cultured articular chondrocytes (Li et al., 2008). Therefore, the effect of HT-2 toxin in inhibiting aggrecan synthesis, promoting aggrecanases, and consequently inducing ECM degradation in chondrocytes is likely associated with an abnormal HA-HC transferring action, which is interesting to elucidate and promote the study of KBD pathogenesis.

## Conclusion

Unlike the chondroprotective function in osteoarthritis, the upregulation of the TSG-6 gene may play an important role in promoting the ECM degradation of KBD chondrocytes. Although it remains unknown whether the pathological HC-HA complex formation process is responsible for TSG-6 induced articular ECM degradation, this gene could be an intermediate factor between HT-2 toxin exposure and chondrocyte injury in KBD. Additionally, an *in vivo* study would make this promising result more convincing.

## Data Availability

The original contributions presented in the study are included in the article/Supplementary Material, and further inquiries can be directed to the corresponding author.
