# Supplementary material for: Abnormal expression of TSG-6 disturbs extracellular matrix homeostasis in chondrocytes from endemic osteoarthritis
Source: Front Genet. 2022 Nov 18;13:1064565. doi: 10.3389/fgene.2022.1064565 (PMC9715581; doi:10.3389/fgene.2022.1064565)
Supplement: Supplementary file 1 [file Table1.DOCX]

Table S1 General Information of subjects included for verification of TSG-6 by RT-PCR and IHC

| No. | KBD group | |  | Normal group | |
| --- | --- | --- | --- | --- | --- |
|  | Age (year) | Gender |  | Age (year) | Gender |
| 1 | 56 | Female |  | 57 | Female |
| 2 | 65 | Female |  | 67 | Female |
| 3 | 46 | Male |  | 45 | Male |
