# Supplementary figures and images for: Abnormal expression of TSG-6 disturbs extracellular matrix homeostasis in chondrocytes from endemic osteoarthritis
Source: Front Genet. 2022 Nov 18;13:1064565. doi: 10.3389/fgene.2022.1064565 (PMC9715581; doi:10.3389/fgene.2022.1064565)

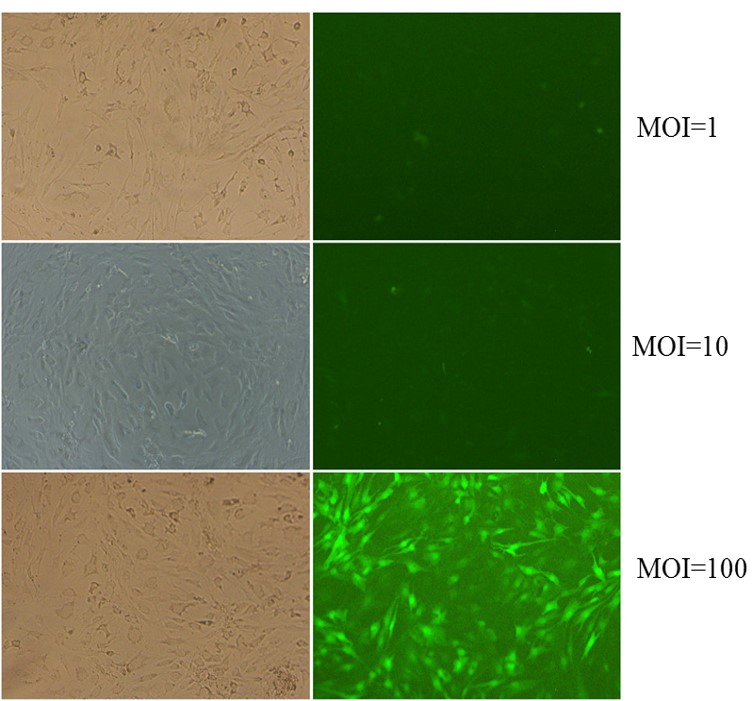

Supplement: Supplementary file 2 [file Image1.JPEG]
