# Supplementary material for: Abnormal expression of TSG-6 disturbs extracellular matrix homeostasis in chondrocytes from endemic osteoarthritis
Source: Front Genet. 2022 Nov 18;13:1064565. doi: 10.3389/fgene.2022.1064565 (PMC9715581; doi:10.3389/fgene.2022.1064565)
Supplement: Supplementary file 4 [file Table2.DOCX]

Table S2 General Information of subjects included for TSG-6 silencing and overexpression

| No. | KBD group | |  | Normal group | |
| --- | --- | --- | --- | --- | --- |
|  | Age (year) | Gender |  | Age (year) | Gender |
| 1 | 56 | Female |  | 57 | Female |
| 2 | 65 | Female |  | 67 | Female |
| 3 | 46 | Male |  | 45 | Male |
| 4 | 64 | Male |  | - | - |
| 5 | 58 | Female |  | - | - |
