# Supplementary material for: Abnormal expression of TSG-6 disturbs extracellular matrix homeostasis in chondrocytes from endemic osteoarthritis
Source: Front Genet. 2022 Nov 18;13:1064565. doi: 10.3389/fgene.2022.1064565 (PMC9715581; doi:10.3389/fgene.2022.1064565)
Supplement: Supplementary file 5 [file Table5.DOCX]

Table S5 Primer information of target genes

| Gene | Forward primer (5’-3’) | Reverse primer (5’-3’) |
| --- | --- | --- |
| MMP1 | ACAACTGCCAAATGGGCTTGA | CTGTCCCTGAACAGCCCAGTAACTTA |
| MMP3 | CTGGGCCAGGGATTAATGGAG | CAATTTCATGAGCAGCAACGAGA |
| MMP13 | TCCCAGGAATTGGTGATAAAGTAGA | GCATGACGCGAACAATACGG |
| COL2A1 | CCAGTTGGGAGTAATGCAAGGA | ACACCAGGTTCACCAGGTTCA |
| Aggrecan | ACAACTGCCAAATGGGCTTGA | CTGTCCCTGAACAGCCCAGTACTTA |
| GAPDH | GCACCGTCAAGGCTGAGAAC | TGGTGAAGACGCCAGTGGA |
