# Supplementary material for: Abnormal expression of TSG-6 disturbs extracellular matrix homeostasis in chondrocytes from endemic osteoarthritis
Source: Front Genet. 2022 Nov 18;13:1064565. doi: 10.3389/fgene.2022.1064565 (PMC9715581; doi:10.3389/fgene.2022.1064565)
Supplement: Supplementary file 6 [file Table3.DOCX]

Table S3 Conditions of MOI selection in pretest for TSG-6 transfection

| Groups | 1×10^8^  MOI=100 | 1×10^7^  MOI=10 | 1×10^6^  MOI=1 |
| --- | --- | --- | --- |
| Control | media:100μl | media:100 μl | media:100 μl |
| A | media:90μl  Virus:10μl | media:90μl  Virus:10μl | media:90μl  Virus:10μl |
| B | media:80μl  P(M):10μl  virus:10μl | media:80μl  P(M):10μl  virus:10μl | media:80μl  P(M):10μl  virus:10μl |
| C | ENi.S:90 μl  Virus:10μl | ENi.S:90 μl  Virus:10μl | ENi.S:90 μl  Virus:10μl |
| D | ENi.S:80μl  P(E):10μl  virus:10μl | ENi.S:80μl  P(E):10μl  virus:10μl | ENi.S:80μl  P(E):10μl  virus:10μl |
