# Supplementary material for: Abnormal expression of TSG-6 disturbs extracellular matrix homeostasis in chondrocytes from endemic osteoarthritis
Source: Front Genet. 2022 Nov 18;13:1064565. doi: 10.3389/fgene.2022.1064565 (PMC9715581; doi:10.3389/fgene.2022.1064565)
Supplement: Supplementary file 7 [file Table4.DOCX]

Table S4 General information of gene sections of TSG-6 for transfection

| NO. | Accession | Target Seq | CDS | | GC(%) |
| --- | --- | --- | --- | --- | --- |
| TSG6-RNAi (54322-1) | NM_007115 | CAAATGAGTACGAAGATAA | | 76.909 | 31.58 |
| TSG6-RNAi (54323-1) | NM_00711 | GGCGTCTTTACAGATCCAA | | 76.909 | 47.3 |
| TSG6-RNAi (54324-11) | NM_00711 | CTAAGGGCAGAGTTGGATA | | 76.909 | 47.3 |
| Description | Human TNF alpha stimulated gene 6 (TSG-6), mRNA. | | | | |
