# Supplementary material for: Abnormal expression of TSG-6 disturbs extracellular matrix homeostasis in chondrocytes from endemic osteoarthritis
Source: Front Genet. 2022 Nov 18;13:1064565. doi: 10.3389/fgene.2022.1064565 (PMC9715581; doi:10.3389/fgene.2022.1064565)
Supplement: Supplementary file 8 [file Table6.DOCX]

Table S5 Differentially expressed genes in KBD chondrocytes compared to normal controls

| Full name of genes | Abbreviation | Public ID | Log2(FC) |
| --- | --- | --- | --- |
| *Upregulated genes* |  |  |  |
| TNF alpha induced protein 6 | TNFAIP6 | NM_007115 | 4.19 |
| guanylate cyclase activator 2A | GUCA2A | NM_033553 | 2.39 |
| cytokine receptor like factor 1 | CRLF1 | NM_004750 | 2.78 |
| potassium channel tetramerization domain containing 1 | KCTD1 | NM_001136205 | 2.42 |
| forkhead box N3 | CHES1(FOXN3) | NM_001085471 | 2.05 |
| fibulin 1 | FBLN1 | NM_001996 | 2.54 |
| ATP binding cassette subfamily C member 8 | ABCC8 | NM_000352 | 2.34 |
| NK2 homeobox 5 | NKX2-5 | NM_001166175 | 1.95 |
| troponin T1, slow skeletal type | TNNT1 | NM_001126132 | 2.17 |
| angiopoietin like 5 | ANGPTL5 | NM_178127 | 2.26 |
| one cut homeobox 2 | ONECUT2 | NM_004852 | 2.35 |
| C-type lectin domain family 3 member B | CLEC3B | NM_001308394 | 1.53 |
| olfactory receptor family 4 subfamily D member 10 | OR4D10 | NM_001004705 | 2.44 |
| TNF receptor superfamily member 11b | TNFRSF11B | NM_002546 | 2.10 |
| fibronectin 1 | FN1 | NM_001306129 | 2.31 |
| *Downregulated genes* |  |  |  |
| 3'-phosphoadenosine 5'-phosphosulfate synthase 2 | PAPSS2 | NM_001015880 | -1.52 |
| frizzled-related protein | FRZB | NM_001463 | -2.34 |
| chordin like 2 | CHRDL2 | NM_001278473 | -2.60 |
| Pituitary tumor-transforming 1 interacting protein | PTTG1IP | NR_104597 | -1.94 |
| ysyl oxidase like 2 | LOXL2 | NM_002318 | -2.43 |
| collagen type XI alpha 1 chain | COL11A1 | NM_001190709 | -2.29 |
| insulin like growth factor binding protein 2 | IGFBP2 | NM_000597 | -1.89 |
| pentraxin 3 | PTX3 | NM_002852 | -1.66 |
| phosphoglycerate kinase 1 | PGK1 | NM_000291 | -1.61 |
| small proline rich protein 2C | SPRR2C | NR_003062 | -1.79 |
| zinc finger DHHC-type containing 13 | ZDHHC13 | NM_001001483 | -2.00 |
| RNA polymerase mitochondrial | POLRMT | NM_005035 | -1.63 |
| thymosin beta 15a | TMSL8 | NM_021992 | -1.85 |
| interleukin 15 receptor subunit alpha | IL15RA | NM_001243539 | -1.68 |
| glyceraldehyde-3-phosphatedehydrogenase | GAPDH | NM_001256799 | -1.77 |
| stathmin 2 | STMN2 | NM_001199214 | -3.01 |
| growth arrest specific 6 | GAS6 | NM_000820 | -1.89 |
| synaptophysin like 2 | SYPL2 | NM_001040709 | -1.77 |
| actin,alpha2,smooth muscle, aorta | ACTA2 | NM_001141945 | -2.72 |
| protease, serine 23 | PRSS23 | NM_001293178 | -1.97 |
| aralkylamine N-acetyltransferase | AANAT | NM_001088 | -1.60 |
| PolypeptideN-acetylgalactosaminyltransferase 1 | GALNT1 | NM_020474 | -2.40 |
| transforming growth factor beta induced | TGFBI | NM_000358 | -1.58 |
| patatin like phospholipase domain containing 4 | PNPLA4 | NM_001142389 | -1.94 |
| CD82 molecule | CD82 | NM_001024844 | -1.76 |
| connective tissue growth factor | CTGF | NM_001901 | -1.67 |
| MAP kinase activating death domain | MADD | NM_001135943 | -1.78 |
| transmembrane protein 109 | TMEM109 | NM_024092 | -1.67 |
| tropomyosin 1 (alpha) | TPM1 | NM_000366 | -2.55 |
| collagen type I alpha 1 chain | COL1A1 | NM_000088 | -1.53 |
| fibronectin 1 | FN1 | NM_001306129 | -2.60 |
| pyridine nucleotide-disulphide oxidoreductase domain 2 | C10orf33 | XM_011540293 | -1.68 |
| solute carrier family 2 member 8 | SLC2A8 | NM_001271711 | -1.79 |
| transcription elongation factor A like 4 | TCEAL4 | NM_001006935 | -1.96 |
| phosphoserine aminotransferase 1 | PSAT1 | NM_021154 | -2.25 |
| Smith-Magenis syndrome chromosome region, candidate 8 | SMCR8 | NM_144775 | -1.62 |
| DC-STAMP domain containing 2 | DCST2 | XM_011509188 | -1.95 |
| calmodulin 2 | CALM2 | NM_001305624 | -1.60 |
| collagen type V alpha 2 chain | COL5A2 | NM_000393 | -2.45 |
| tropomyosin 1 (alpha) | TPM1 | NM_000366 | -2.66 |
| VPS18, CORVET/HOPS core subunit | VPS18 | NM_020857 | -1.53 |
| mitogen-activated protein kinase 3 | MAPK3 | NM_001040056 | -1.61 |
| insulin like growth factor binding protein 4 | IGFBP4 | NM_001552 | -1.85 |
| caldesmon 1 | CALD1 | NM_004342 | -2.19 |
| actin gamma 1 | ACTG1 | NM_001199954 | -2.18 |
| lactate dehydrogenase A | LDHA | NM_001135239 | -1.88 |
| proline-serine-threonine phosphatase interacting protein 1 | PSTPIP1 | NM_001321135 | -2.07 |
| zinc finger protein 142 | ZNF142 | NM_001105537 | -2.01 |
| inhibitor of DNA binding 4, HLH protein | ID4 | NC_000006 | -2.98 |
| polyglutamine binding protein 1 | PQBP1 | NM_001032381 | -1.64 |
| SPARC related modular calcium binding 2 | SMOC2 | NM_001166412 | -2.05 |
| C-X-C motif chemokine ligand 1 | CXCL1 | NM_001511 | -2.25 |
| TNF receptor superfamily member 11b | TNFRSF11B | NM_002546 | -2.33 |
| hyaluronan and proteoglycan link protein 1 | HAPLN1 | NM_001884 | -2.30 |
| X-prolyl aminopeptidase 2 | XPNPEP2 | NM_003399 | -1.60 |
